# Impact of Non-alcoholic Fatty Liver Disease on long-term cardiovascular events and death in Chronic Obstructive Pulmonary Disease

**DOI:** 10.1038/s41598-018-34988-2

**Published:** 2018-11-08

**Authors:** Damien Viglino, Anais Plazanet, Sebastien Bailly, Meriem Benmerad, Ingrid Jullian-Desayes, Renaud Tamisier, Vincent Leroy, Jean-Pierre Zarski, Maxime Maignan, Marie Joyeux-Faure, Jean-Louis Pépin

**Affiliations:** 1 Emergency Department, Grenoble Alpes University Hospital, Grenoble, France; 2 HP2 laboratory, INSERM U1042, University Grenoble Alpes, Grenoble, France; 3 EFCR Laboratory, Pole Thorax et Vaisseaux, Grenoble Alpes University Hospital, Grenoble, France; 4 Hepatogastroenterology Department, Grenoble Alpes University Hospital, Grenoble, France; 5 INSERM U823, IAPC Institute for Advanced Biosciences, University Grenoble Alpes, Grenoble, France

## Abstract

Chronic Obstructive Pulmonary Disease (COPD) and Non-Alcoholic Fatty Liver Disease (NAFLD) both independently increase cardiovascular risk. We hypothesized that NAFLD might increase the incidence of cardiovascular disease and death in COPD patients. The relationship between NAFLD, incident cardiovascular events, and death was assessed in a prospective cohort of COPD patients with 5-year follow-up. Noninvasive algorithms combining biological parameters (FibroMax^®^) were used to evaluate steatosis, non-alcoholic steatohepatitis (NASH) and liver fibrosis. Univariate and multivariate Cox regression models were used to assess the hazard for composite outcome at the endpoint (death or cardiovascular event) for each liver pathology. In 111 COPD patients, 75% exhibited liver damage with a prevalence of steatosis, NASH and fibrosis of 41%, 37% and 61%, respectively. During 5-year follow-up, 31 experienced at least one cardiovascular event and 7 died. In univariate analysis, patients with liver fibrosis had more cardiovascular events and higher mortality (Hazard ratio [95% CI]: 2.75 [1.26; 6.03]) than those with no fibrosis; this remained significant in multivariate analysis (Hazard ratio [95% CI]: 2.94 [1.18; 7.33]). We also found that steatosis and NASH were not associated with increased cardiovascular events or mortality. To conclude, early assessment of liver damage might participate to improve cardiovascular outcomes in COPD patients.

## Introduction

Chronic obstructive pulmonary disease (COPD) is a growing public health concern, causing considerable health-related costs and increased premature mortality. Although diagnosis is mainly based on chronic airflow limitation, as assessed by post-bronchodilator spirometry, COPD is nowadays considered a complex, heterogeneous and multi-organ condition. It is increasingly recognized that the presence of comorbidities such as obesity and/or cardiovascular (CV) and metabolic diseases substantially contributes to the recurrence of hospitalization for exacerbation and significantly impacts prognosis and the incidence of late CV events^1–3^. Recent literature and the Global initiative for chronic Obstructive Lung Disease (GOLD) recommendations emphasize the importance of an integrated approach to COPD with a careful assessment and treatment of comorbidities^3,4^. While CV comorbidities^5^ and type 2 diabetes^6^ have been extensively described in COPD patients, there is an emerging interest regarding liver damage and Non-alcoholic Fatty Liver Disease (NAFLD) in COPD patients^7^.

NAFLD is characterized by the accumulation of hepatic triglycerides (steatosis) in patients without significant alcohol consumption or viral hepatitis^8^. Some patients have an additional inflammatory infiltration (nonalcoholic steatohepatitis (NASH)) and can eventually develop liver fibrosis leading to an increased risk of hepatocellular carcinoma and/or cirrhosis^9,10^.

COPD and NAFLD share common risk factors, such as reduced physical activity, and pathophysiological mechanisms including oxidative stress, low grade inflammation, and metabolic syndrome^11^. We recently showed that the prevalence of NAFLD is elevated in COPD patients, with 41% of liver steatosis, 37% of NASH and 61% of liver fibrosis^7^. COPD severity is also recognized as an independent risk factor of NAFLD^7^. Compelling evidence over the past several years has substantiated a significant link between NAFLD and CV diseases^8,12–22^. We hypothesized that NAFLD might increase the incidence of CV diseases and death in COPD patients. Therefore, the relationship between NAFLD, incident cardiovascular events, and death was assessed in a prospective cohort of COPD patients with 5-year follow-up.

## Material and Methods

### Study population

A prospective COPD cohort with associated biobanking was initiated in 2007 in Grenoble Alps University Hospital having been approved by an independent ethics committee (Comité de Protection des Personnes Sud Est V, IRB0006705 10/01/2007). All methods were performed in accordance with the relevant guidelines and regulations. Patients aged 18 years or over, with documented COPD or Forced Expiratory Volume in one second/Forced Vital Capacity (FEV_1_/FVC) <70% were eligible. Patients with active pulmonary infection, chronic heart failure, left ventricular ejection fraction <45%, active smoking >10 cigarettes per day, neoplasia, antioxidant treatment (such as N-acetylcysteine, selenium, vitamin C, vitamin E), and pregnant women were excluded. Also those with a daily consumption of alcohol ≥20g for women and ≥30 g for men and those with viral hepatitis were excluded as these are confounding factors for liver diseases. All patients have given their informed consent.

### Procedures

Comorbidities were assessed using predefined cut-offs to objective clinical measurements and laboratory analyses (see later)^23^.

At study enrolment, fasting serum samples were collected from all patients, frozen and stored at −80 °C until analyses were conducted.

### Metabolic parameters

Ten biochemical markers, including liver enzymes, lipid profile, fasting glycaemia and insulin were measured in order to characterize metabolic comorbidities and calculate FibroMax® scores: α2-macroglobulin, apolipoprotein A1, haptoglobin, γ-glutamyltransferase (GGT), total bilirubin, alanine aminotransferase (ALT), aspartate aminotransferase (AST), fasting blood glucose, triglycerides and total cholesterol. Measurements were performed with *D*imension Vista (Siemens©) and CE-IVD kits.

Anonymized data were sent to the calculation centre (BioPredictive, Paris, France) to obtain FibroMax® scores blinded to the severity of COPD and comorbidities. Insulin resistance was assessed using the homeostatic model assessment for insulin resistance (HOMA-IR), calculated using the following formula: insulinaemia × glucose/22.5 (glucose units mmol.L^−1^)^24^.

### FibroMax algorithm

The Fibromax® algorithm was used to noninvasively evaluate liver damage as previously described^7^. It combines three tests: FibroTest (FT), SteatoTest (ST) and the NashTest (NT)^25,26^. FT includes α2-macroglobulin, apolipoprotein A1, haptoglobin, GGT and total bilirubin values, age and sex, and provides a quantitative estimate ranging from 0.00 to 1.00, with higher values corresponding to a greater probability of lesions. Scores correspond to the METAVIR stages^27^ as follows: F0 (0.00–0.21); F0–F1 (0.22–0.27); F1 (0.28–0.31); F1–F2 (0.32–0.48); F2 (0.49–0.58); F3 (0.59–0.72); F3–F4 (0.73–0.74) and F4 (0.75–1.0) [19]. ST combines FT parameters with height and weight plus ALT, fasting serum glucose, triglycerides and cholesterol. ST scores range from 0.00 to 1.00 used to attribute four steatosis stages from S0 to S4, as follows, S0 (0.00–0.37): no steatosis; S1 (0.38–0.56): minimal steatosis <5%; S2 (0.57–0.68): moderate steatosis 6–32%; and S3–S4 (0.69–1.0): severe steatosis >32%. Finally, the NT score is calculated from weight, height, AST, fasting serum glucose, triglycerides and cholesterol in addition to age, sex and FT components. The NT distinguishes three NASH categories: N0 (0.00–0.25) for absence of NASH; N1 (>0.25–0.50) for borderline NASH; and N2 (>0.50–0.75) for NASH [18,19]. FT ≥ F0-F1, ST ≥ S2 and NT ≥ N1 were considered as positives, as previously applied by Minville *et al*. and Viglino *et al*.^7,28^.

### Patient follow-up and outcomes

Patients included in the cohort were systematically followed-up at 1, 2, 3 and 5 years. At each visit, patients were questioned regarding the occurrence of incident CV events and AECOPD since the last visit. Incident CV events were defined by the new occurrence of a cardiac event during follow-up: acute myocardial infarction; stroke; new diagnosis of peripheral arterial disease or acute limb ischemia; venous thromboembolic disease and/or pulmonary embolism and new onset arrhythmias. When necessary complementary information was obtained through electronic medical records from Grenoble Alps University Hospital and the patient’s general practitioner was phoned to collect additional information. Cardiovascular events were defined as myocardial infarction, stroke, peripheral arterial disease, pulmonary embolism, atrial fibrillation, and acute pulmonary edema and were validated by the two first authors.

### Data analysis

#### Population characteristics

Qualitative variables are presented in numbers and percentages and quantitative variables in median and interquartile range. Comparison of the variables was performed separately for NASH, fibrosis and steatosis using a Mann-Whitney test for quantitative variables and a Chi-square test or an exact Fisher test for qualitative variables.

#### Relationship between liver diseases assessed by Fibromax® and CV events and death during 5 years of longitudinal follow-up

First a univariate survival analysis was performed using the Kaplan-Meier estimator and the Log-Rank test to assess the impact of the presence of a liver disease on the composite outcomes. The Kaplan-Meier analysis was also performed to assess separately the impact of each liver disease on the composite outcome. The results were reported by using the Kaplan-Meier curves and the Log-rank p-value.

In a second time, to identify the factors associated with the 5-year composite outcome, a univariate and a multivariate Cox regression model were performed. Univariate Cox models were performed to select variables associated with the 5-year composite outcome with a p-value threshold of 0.20. Selected variables were introduced into the multivariable Cox model including age and sex as confounding factors. For all Cox models, the proportionality hazard assumption was tested and verified, and log-linearity of continuous variables was also tested. Statistical analyses were performed using SAS v9.4 (SAS Institute Inc., Cary, NC, USA). A p-value <0.05 was considered as significant.

## Results

**Figure 1:**
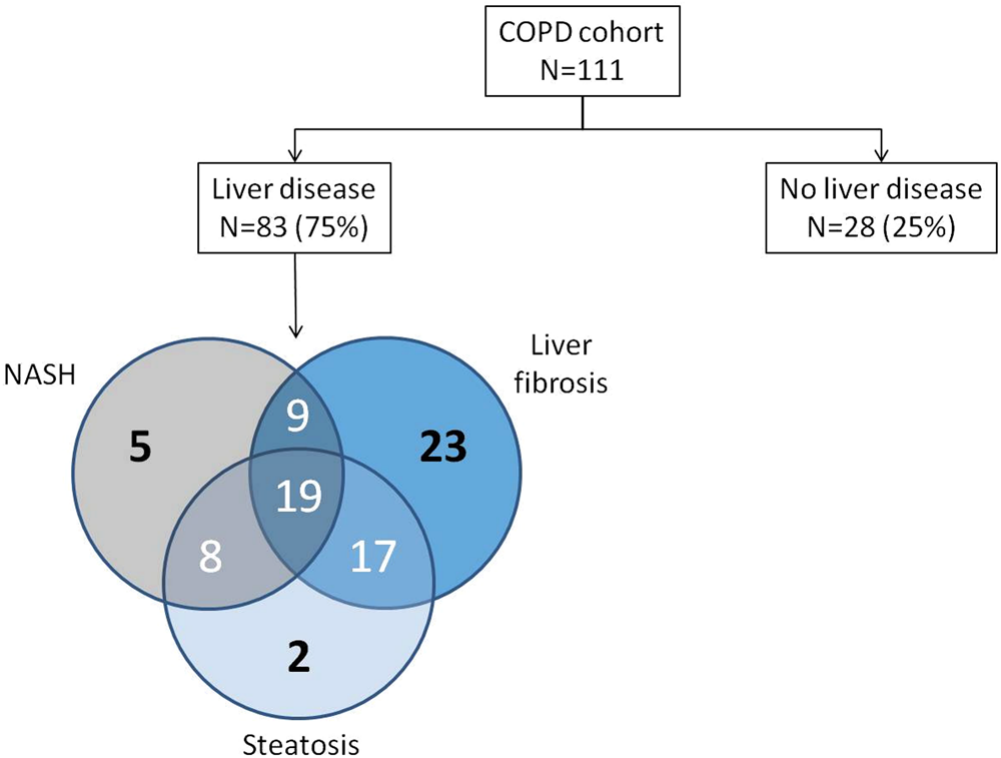
Prevalence of liver diseases observed in COPD patients at baseline. Main result: 83/111 COPD patients exhibit liver diseases.

A cohort of 111 COPD patients (described in Tables 1 and S1 of the supplementary file) was analyzed. They were predominantly men (78%), aged of 64 [59; 70] years (median [25^th^; 75th percentiles]), with a body mass index (BMI) of 26 [22; 28] kg/m^2^. At inclusion, 28 patients (25%) had no liver disease and 83 (75%) had at least one type of liver disease (Fig. 1). Forty six patients (41%) had moderate to severe steatosis, 41 (37%) exhibited NASH, and 68 (61%) presented liver fibrosis.

**Table 1:** Characteristics of the studied population at inclusion in the cohort.

	No liver disease n = 28	Any liver disease n = 83	*p* ^*^
Male	15 (53.6)	71 (85.5)	<0.01
Age (years)	55.4 [51; 64.8]	65.3 [61.1; 70.9]	<0.01
BMI (kg/m²)	22.2 [19.8; 23.6]	26.5 [23.6; 28.7]	<0.01
Smoking (pack year)	32.5 [9.6; 40.3]	41 [22; 50]	0.11
OSA			0.03
Untreated	4 (14.8)	33 (40.7)	
Treated	2 (7.4)	8 (9.9)	
No	21 (77.8)	40 (49.4)	
Hypertension	9 (32.1)	44 (53)	0.06
Dyslipidemia	6 (21.4)	39 (47)	0.02
Type 2 diabetes	1 (3.6)	16 (19.3)	0.05
HOMA	1.6 [0.8; 3.2]	2.1 [1; 5.1]	0.22
FEV1 (%)	67.5 [53.5; 88.5]	64 [53; 76]	0.19
FEV1/FVC (%)	53.5 [44.5; 67.5]	57 [49; 65]	0.94
GOLD			0.07
1	8 (28.6)	9 (10.8)	
2	13 (46.4)	53 (63.9)	
3–4	7 (25)	21 (25.3)	

BMI, body mass index; FEV_1,_ Forced Expiratory Volume in one second; FVC, Forced Vital Capacity; HOMA, Homeostasis model assessment of insulin resistance; OSA, obstructive sleep apnea.

Data are expressed as n (%) or median and IQR. Comparison of the variables was performed using a Mann-Whitney test for quantitative variables and a Chi square test or an exact Fisher test for qualitative variables.

^*^Data from patients with no liver disease compared to those with any liver disease.

### Relationship between liver damage assessed by Fibromax^®^ and CV events and death during the 5 years of longitudinal follow-up

During the follow-up, 56 (50%) had at least one exacerbation, 31 (28%) experienced at least one late CV event and 7 (6%) patients died (Table 2). One patient developed cirrhosis and two patients developed hepatocellular carcinoma. The number of hospitalizations after exacerbation was higher in COPD patients with liver disease compared to those with no liver disease (Table 2). Deaths were mainly of pulmonary origin (three lung cancers, two respiratory insufficiencies, one pulmonary embolism and one of unknown cause).

**Table 2:** Description of exacerbations, CV events, liver disease evolution and death for patients with and without liver disease.

	No liver disease n = 28	Any liver disease n = 83	*p* ^*^
**Cardiovascular events**	4 (14.3)	27 (32.5)	0.09
Myocardial infarction	0 (0)	1 (1.2)	1
Stroke	0 (0)	2 (2.4)	1
Peripheral arterial disease	4 (14.3)	19 (22.9)	0.42
VTD	1 (3.6)	0 (0)	0.25
Arrhythmias	2 (7.1)	8 (9.6)	1
**Exacerbations**	13 (46.4)	38 (45.8)	0.95
Emergency admission	7 (25)	33 (39.8)	0.16
Hospitalization	12 (42.9)	57 (68.7)	0.01
Intensive care unit and resuscitation	4 (14.3)	12 (14.5)	1
**Death**	0 (0)	7 (8.4)	0.19
Lung cancer	0 (0)	3 (3.6)	0.57
Respiratory failure	0 (0)	2 (2.4)	1
Pulmonary Embolism	0 (0)	1 (1.2)	1
Unknown	0 (0)	1 (1.2)	1

VTD, venous thromboembolic disease.

Data are expressed as n (%).

^*^Data from patients with no liver disease compared to those with any liver disease.

#### Univariate analysis

**Figure 2:**
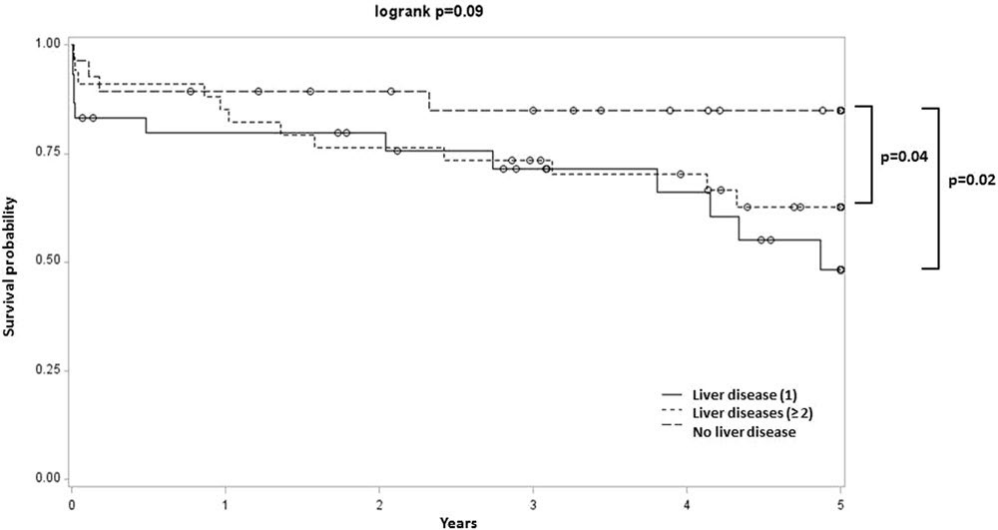
Kaplan-Meier analysis of first CV event and death in COPD patients with at least one liver disease compared to those with no liver disease. Main result: Higher incidence of first CV event and death in COPD patients with at least one liver disease compared to those with no liver disease.

**Figure 3:**
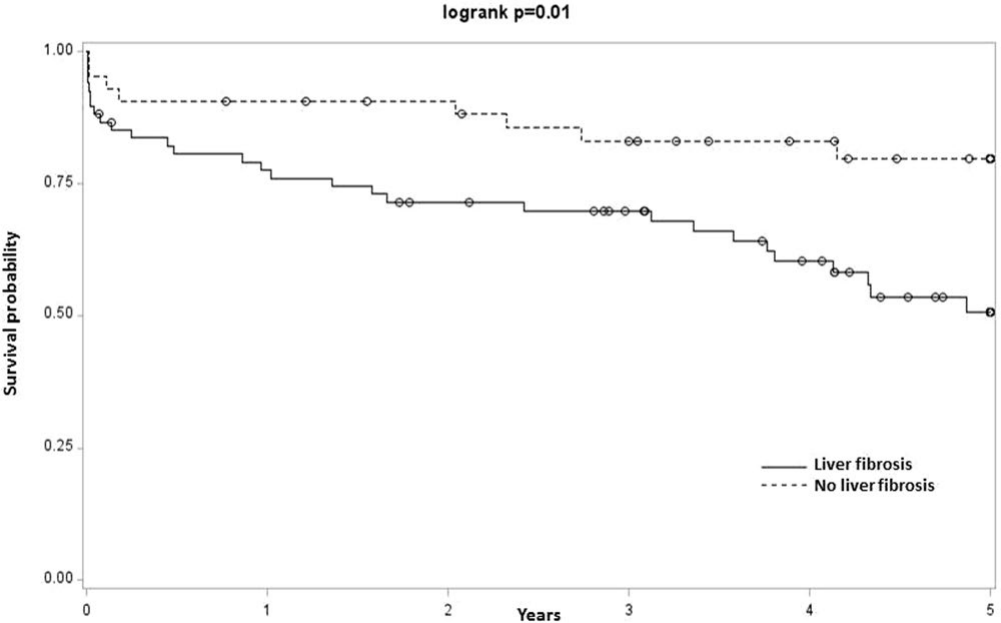
Kaplan-Meier analysis of first CV event and death in COPD patients depending on the presence of fibrosis. Main results: Higher incidence of first CV event and death in COPD patients with fibrosis compared to those with no fibrosis.

The risk of CV events and mortality was higher in patients with at least one liver disease (Log-rank test, p = 0.03) than for those with no liver disease (Fig. 2). In patients with fibrosis, the rate of CV events and mortality was significantly higher compared to COPD patients without liver fibrosis (Hazard ratio [95% CI]: 2.75 [1.26; 6.03], Tables 3, S2 and Fig. 3). Steatosis and NASH were not associated with an increased rate of CV events or mortality (Table 3, Figs S1 and S2). Moreover, none of the liver diseases was associated with an increased rate of AECOPDs (Table 3).

**Table 3:** Description of exacerbations and CV events according to the different types of liver disease.

	General population n = 111	Steatosis n = 46	p*	NASH n = 41	*p*°	Fibrosis n = 68	*p*‘
**Cardiovascular events**	31 (28)	14 (30.4)	0.62	13 (31.7)	0.48	23 (33.8)	0.08
Myocardial infarction	1 (0.9)	0 (0)	1	1 (2.4)	0.37	1 (1.5)	1
Stroke	2 (1.8)	0 (0)	0.51	1 (2.4)	1	1 (1.5)	1
Peripheral arterial disease	23 (20.7)	10 (21.7)	0.82	11 (26.8)	0.22	15 (23.5)	0.66
VTD	1 (0.9)	0 (0)	1	0 (0)	1	0 (0)	0.39
Arrhythmias	10 (9.0)	6 (13.0)	0.31	2 (4.9)	0.32	8 (11.8)	0.31
**Exacerbation**	51 (45.9)	24 (52.2)	0.27	16 (39)	0.26	30 (44.1)	0.63
Emergency admission	40 (36.0)	16 (34.8)	0.82	16 (39)	0.62	26 (38.2)	0.54
Hospitalization	69 (62.2)	32 (69.6)	0.18	25 (61)	0.84	47 (69.1)	0.06
Intensive care unit and resuscitation	16 (14.4)	8 (17.4)	0.45	7 (17.1)	0.54	9 (13.2)	0.66
**Death**	7 (6.3)	5 (10.9)	0.12	3 (7.3)	0.71	7 (10.3)	**0**.**04**

NASH, Non-Alcoholic Steato-Hepatitis; VTD, venous thromboembolic disease.

Data are expressed as n (%).

^*^Data from patients with a positive Steatotest^®^ compared to those with a negative Steatotest^®^ in univariate analysis.

^°^Data from patients with a positive Nashtest^®^ compared to those with a negative Nashtest^®^ in univariate analysis

^‘^Data from patients with a positive Fibrotest^®^ compared to those with a negative Fibrotest^®^ in univariate analysis.

#### Multivariate analysis

After adjustment on age, BMI, gender, inhaled corticosteroids, dyslipidemia and diabetes, fibrosis remained a significant risk factor for CV events and death (HR: 2.94 [1.18; 7.33], p = 0.02, details of HR are presented in Table S3 of supplementary file).

## Discussion

In this prospective cohort, COPD patients with positive biomarkers of liver disease had an increased 5-year risk of CV events. Liver fibrosis in particular was associated with a higher 5-year risk of CV events and death. Liver disease did not impact the rate of COPD exacerbations.

Over the past decade it has been shown that NAFLD is strongly associated with an increased risk of CV diseases, which are the leading cause of mortality among NAFLD patients^9,29,30^. A meta-analysis concluded that the presence of NAFLD (diagnosed by imaging or histology) is associated with an increased probability of fatal and non-fatal incident CV events (OR: 1.64, 95% CI: [1.26; 2.13])^31^. This risk appears to further increase with NAFLD severity; as in this meta-analysis fibrosis appears to have a more severe impact on the risk of CV events and death than other NAFLD subtypes (OR: 1.94, 95% CI: [1.17; 3.21]). Our results in a COPD population are fully consistent with these data, showing an even higher risk (HR: 2.94, 95% CI: [1.18; 7.33]) and supporting an elevated risk in cases with combined COPD-NAFLD. Our results are in line with a longitudinal multicenter prospective study of 859 patients with NAFLD, in which liver fibrosis was independently associated with long-term overall mortality (the leading cause of death was CV diseases, at 38%) whereas other histologic features of steatohepatitis were not^17^. Another cohort study found that liver fibrosis predicted overall and disease-specific mortality^32^.

Previous studies have tried to identify different phenotypes of COPD patients: frequent exacerbators^31–36^, eosinophilic patients and those with asthma-COPD overlap syndrome^37,38^, and patients with severe airflow limitation or patients with milder airflow limitation but with obesity and CV comorbidities^39^. The identification of such phenotypes may have an impact both on long term integrated care and exacerbations^40,41^. The identification of comorbidities is a key step in characterizing these phenotypes^1^. Interestingly, the NAFLD patients in our cohort were older, they were more often men, and they had more other CV risk factors, although their FEV_1_ at baseline was not different from that of COPD patients without NAFLD. These NAFLD patients have characteristics very similar to those of COPD patients with an increased risk of myocardial infarction^42,43^.

This study had some limitations. Indeed, although it is a well phenotyped prospective cohort, there was no information about time-dependent additional confounders which might have impact the outcomes. Moreover, the relatively limited sample size might under-powered the assessment of liver diseases outcomes. No definite relationship between observed deaths and NAFLD severity can be established owing to the lack of power for this specific type of events. However, we included deaths in the analysis of composite “cardiovascular events and death” as is usually the case in studies addressing hard outcomes in the cardiovascular field. According to these limitations, our results should be confirmed by external validation. However, we strongly suggest that NAFLD should be systematically investigated in COPD patients as a significant contributing factor to CV morbi-mortality. We acknowledge that the ideal design to study the potential cumulative effects of NAFLD and COPD would have been to provide information in 4 conditions: COPD and healthy patients, patients presenting with NAFLD and not in each of these groups. However, it is nearly impossible to achieve an appropriate matching for confounders in these populations. The effect of liver damage in COPD is a very novel area of research and we think that our data really represent an original dataset interesting to share with the scientific community. Our current objective was to demonstrate the association in COPD population with NAFLD and cardiovascular disease. The second step will be intervention studies.

The diagnosis of NAFLD in COPD could have therapeutic implications. Specific and non-specific treatments for NAFLD are currently being evaluated and subgroup analyses of COPD patients may be of interest in order to explore the possibilities for reducing the risk of CV events^44,45^.

## Conclusion

This longitudinal prospective study was the first attempt to evaluate the impact of NAFLD on the incidence of cardiovascular events and death in COPD patients. COPD patients suffering from liver fibrosis exhibited a higher risk of incident CV events. This risk appears greater than in the general hepatic fibrosis population. These results suggest that liver disease should be considered when planning the integrative care of COPD patients.

## Electronic supplementary material

Supplementary materials
