# Supplementary material for: Impact of Non-alcoholic Fatty Liver Disease on long-term cardiovascular events and death in Chronic Obstructive Pulmonary Disease
Source: Sci Rep. 2018 Nov 8;8:16559. doi: 10.1038/s41598-018-34988-2 (PMC6224555; doi:10.1038/s41598-018-34988-2)

Supplementary material

Impact of Non-alcoholic Fatty Liver Disease on long-term cardiovascular events and death in Chronic Obstructive Pulmonary Disease

Damien Viglino[1,2§], Anais Plazanet[1,2§], Sebastien Bailly[2,3], Meriem Benmerad [2,3], Ingrid Jullian-Desayes[2,3], Renaud Tamisier[2,3], Vincent Leroy[4,5], Jean-Pierre Zarski[4,5], Maxime Maignan[1,2], Marie Joyeux-Faure[2,3], Jean-Louis Pépin[2,3*]

1. Emergency Department, Grenoble Alpes University Hospital, Grenoble, France
2. HP2 laboratory, INSERM U1042, University Grenoble Alpes, Grenoble, France
3. EFCR Laboratory, Pole Thorax et Vaisseaux, Grenoble Alpes University Hospital, Grenoble, France
4. Hepatogastroenterology Department, Grenoble Alpes University Hospital, Grenoble, France
5. INSERM U823, IAPC Institute for Advanced Biosciences, University Grenoble Alpes, Grenoble, France

§These two authors equally contributed to the work

***Corresponding author**

Jean-Louis Pépin
EFCR Laboratory, Pole Thorax et Vaisseaux
CHU Grenoble Alpes
CS 10217
38043 Grenoble Cedex 9, France
JPepin@chu-grenoble.fr

Table S1. Characteristics of whole population and each liver disease subgroup

	**Global population n = 111**	**Steatosis n = 46**	***p****	**NASH n = 41**	***p*°**	**Fibrosis n = 68**	***p*'**
Anthropometric data							
Male gender	86 (78)	43 (94)	<0.01	35 (85)	0.13	60 (88)	<0.01
Age (years)	64 [59;70]	65 [62;70]	0.04	66 [62;71]	0.01	65 [61;72]	<0.01
BMI (kg/m^2)	26 [22;28]	28 [26;30]	<0.01	28 [27;29]	<0.01	26 [22 ; 29]	0.08
Smoking (Pack years)	37 [20;50]	41 [26;57]	0.05	35 [20;48]	0.67	43 [20;53]	0.18
OSA			<0.01		0.50		<0.01
	37 (34)	23 (50)		15 (38)		30 (46)	
	10 (9)	7 (15)		5 (12)		5 (8)	
	61 (57)	16 (35)		20 (50)		31 (47)	
Hypertension	53 (48)	26 (57)	0.12	23 (56)	0.18	36 (53)	0.17
Dyslipidemia	45 (41)	24 (52)	0.04	22 (54)	0.03	31 (46)	0.17
Type 2 diabetes	17 (15)	10 (22)	0.11	8 (20)	0.35	15 (22)	0.01
HOMA	2.0 [0.9 ; 4.3]	2.7 [1.7;8.7]	<0.01	2.7 [1.3 ; 5.4]	0.04	2.4 [1.0 ; 5.3]	0.11
Pulmonary function							
FEV_1 (%)	66 [53 ; 80]	62.5 [50 ; 72]	0.07	67 [55 ; 73]	0.80	64 [53.5 ; 78]	0.62
FEV_1/FVC (%)	57 [48 ; 65]	55.5 [50 ; 64]	0.87	58 [50 ; 67]	0.16	58.5 [48.5 ; 65]	0.49
Gold			0.02		0.78		0.29
1	17 (15)	2 (4)		5 (12)		8 (12)	
2	66 (60)	31 (67)		25 (61)		44 (65)	
3-4	28 (25)	13 (28)		11 (27)		16 (24)	

* data from patients with a positive steatotest® compared to those with a negative steatotest® in univariate analysis

° data from patients with a positive nashtest® compared to those with a negative nashtest® in univariate analysis

' data from patients with a positive fibrotest® compared to those with a negative fibrotest® in univariate analysis

BMI, body mass index; OSA, obstructive sleep apnea; HOMA, Homeostasic model assessment of insulin resistance; FEV1/FVC, Forced Expiratory Volume in one second / Force Vital Capacity. Data are expressed as N (%) or median and IQR.

Table S2. Univariate Cox regression for 5-year composite outcome

	HR, CI95%	p
NASH	1.23 [0.64 ; 2.37]	0.527
Steatosis	1.23 [0.65 ; 2.35]	0.522
Fibrosis	2.75 [1.26 ; 6.03]	**0.011**
Liver disease	3.06 [1.08 ; 8.63]	**0.035**
Sex (female)	1.41 [0.68 ; 2.91]	0.353
Age (years)	1.04 [0.99 ; 1.08]	0.089
BMI (kg/m²)	1.03 [0.96 ; 1.1]	0.464
Inhaled corticosteroïds (Yes)	0.49 [0.26 ; 0.94]	**0.032**
Diabetes (Yes)	2.04 [0.96 ; 4.33]	0.063
Hypertension(Yes)	1.41 [0.73 ; 2.7]	0.303
Dyslipidemia (Yes)	1.69 [0.89 ; 3.22]	0.11
Insulinemia	1 [0.98 ; 1.02]	0.836
Glycemia	1.04 [0.92 ; 1.19]	0.534
HOMA	1 [0.93 ; 1.07]	0.903
FEV_1 (%)	1.01 [1 ; 1.03]	0.107
FEV_1/FVC (%)	1.02 [0.99 ; 1.04]	0.13
COPD stage (GOLD)		0.362
2	0.66 [0.28 ; 1.53]	
3-4	0.47 [0.16 ; 1.33]	
1	1	
CRP	1.03 [1 ; 1.07]	0.079
OSA		0.572
Untreated	0.99 [0.5 ; 1.97]	
Treated	0.46 [0.11 ; 1.97]	
No	1	

BMI, body mass index; HOMA, Homeostasic model assessment of insulin resistance; NASH, nonalcoholic steatohepatitis; FEV1/FVC, Forced Expiratory Volume in one second / Force Vital Capacity.

Table S3. Results of multivariable analysis – Cox model for CV events and death at 5-years

	**Steatosis (n=46)**		**NASH (n=41)**		**Fibrosis (n=68)**	
	HR, CI95%	P	HR, CI95%	P	HR, CI95%	P
**Liver disease (NASH, Fibrosis or steatosis)**	1.66 [0.72 ; 3.84]	0.236	0.80 [0.34 ; 1.85]	0.596	2.94 [1.18 ; 7.33]	**0.02**
**Age**		0.061		0.071		0.154
[58.6 ; 64.1[	0.48 [0.16 ; 1.47]		0.48 [0.16 ; 1.47]		0.38 [0.12 ; 1.20]	
[64.1 ; 69.6[	0.72 [0.22 ; 2.31]		0.71 [0.22 ; 2.26]		0.52 [0.15 ; 1.80]	
[69.6 ; 81.0]	1.94 [0.63 ; 6.01]		1.92 [0.61 ; 6.03]		1.11 [0.34 ; 3.62]	
[35.0 ; 58.6[	1		1		1	
**Sex (female)**	2.19 [0.81 ; 5.96]	0.124	1.83 [0.71 ; 4.75]	0.211	1.94 [0.72 ; 5.21]	0.188
**Inhaled corticosteroids**	0.35 [0.16 ; 0.73]	**0.005**	0.36 [0.17 ; 0.75]	**0.006**	0.42 [0.20 ; 0.89]	**0.024**
**BMI**		0.114		0.098		0.089
[20-25[	2.69 [0.57 ; 12.81]		2.69 [0.56 ; 12.88]		2.93 [0.60 ; 14.32]	
[25-30[	2.97 [0.63 ; 14.07]		4.32 [0.87 ; 21.52]		3.54 [0.79 ; 15.91]	
>=30	0.60 [0.07 ; 5.17]		1.06 [0.12 ; 9.23]		0.74 [0.09 ; 5.98]	
<20	1		1		1	
**Diabetes**	1.95 [0.86 ; 4.41]	0.109	1.8 [0.79 ; 4.09]	0.16	1.64 [0.72 ; 3.73]	0.238
**Dyslipidemia**	2.06 [1.01 ; 4.21]	**0.047**	2.12 [1.05 ; 4.28]	**0.036**	1.76 [0.86 ; 3.60]	0.124

BMI, body mass index.

Figure S1. Kaplan Meyer analysis of first CV event and death in COPD patients depending on the presence or not of steatosis

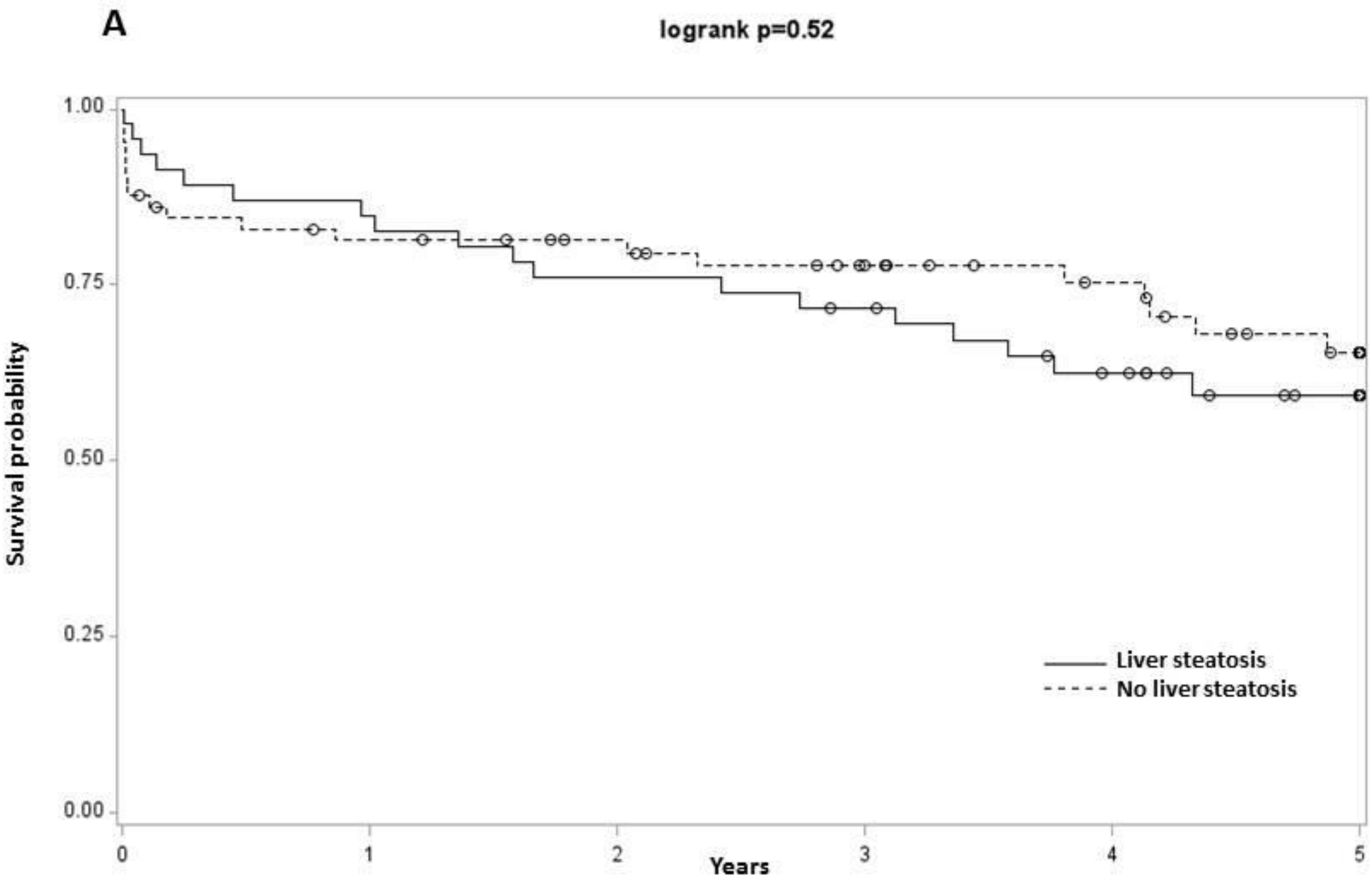

Figure S2. Kaplan Meyer analysis of first CV event and death in COPD patients depending on the presence or not of NASH

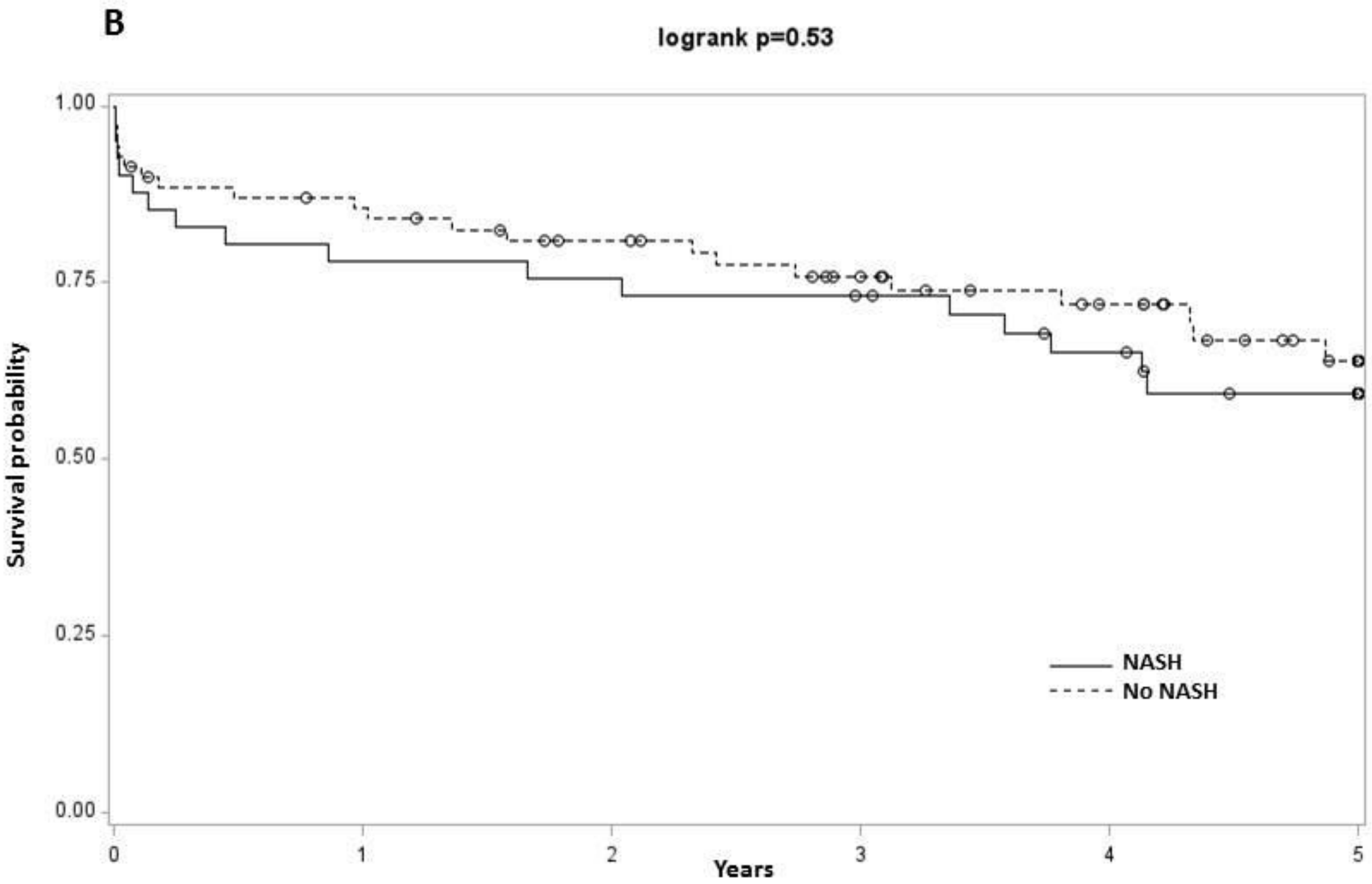

Supplement: Supplementary file 1 — Supplementary materials [file 41598_2018_34988_MOESM1_ESM.pdf]
